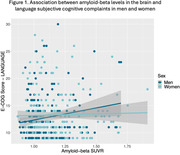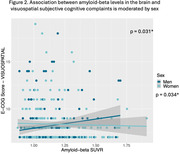# Sex differences in the association between pathology and subjective cognitive complaints in the early stages of Alzheimer’s disease

**DOI:** 10.1002/alz.094802

**Published:** 2025-01-09

**Authors:** Sophie Boutin, Simona Maria Brambati

**Affiliations:** ^1^ Université de Montréal, Montréal, QC Canada; ^2^ Centre de Recherche Institut Universitaire de Gériatrie de Montréal, Montréal, QC Canada

## Abstract

**Background:**

In the preclinical phase of Alzheimer’s disease (AD), individuals deemed at risk exhibit cerebral beta‐amyloid (Aβ) proteins without cognitive impairment, whereas those in the mild cognitive impairment stage display cognitive deficits. Subjective cognitive complaints, or perceived cognitive alterations by the individual without objective decline, emerge during these phases and may correlate with Aβ levels. The assessment of cognitive complaints could thus help identify those vulnerable to AD. Despite increasing interest in the role of biological sex in AD manifestation, it remains less clear in its early stages.

**Method:**

A total of 418 cognitively healthy older subjects and 424 subjects in the mild cognitive impairment stage from the Alzheimer’s Disease Neuroimaging Initiative cohort with PET‐Aβ levels above and below the 1.11 threshold were included in the study. Using ANOVA models, we examined sex differences in cognitive complaints in different domains, i.e., memory, language, attention, organization, planning, visuospatial, assessed using the Everyday Cognition questionnaire. We also tested, using general linear models, whether sex moderated the association between Aβ levels and the severity of complaints in the same groups. Finally, we realized separate logistic regressions in men and women to determine whether the types of cognitive complaints that can predict Aβ positivity vary between sexes at different stages of the disease. In all models, we controlled for age, years of education, and depression and anxiety symptoms.

**Results:**

Although there were no sex differences in complaint types, there was a significant correlation between higher Aβ levels and exacerbated visuospatial and language complaints in both cognitively healthy men and women. Interestingly, this association with visuospatial complaints was stronger in men compared to women. At the stage of mild cognitive impairment only, specific memory, language, and organization complaints predicted Aβ positivity exclusively in women.

**Conclusion:**

These findings suggest that subjective cognitive complaints could be good indicators of increased risk of AD because of their association with one of its biomarkers. Biological sex also seems to exert distinct impacts on AD’s clinical manifestation at different stages of the disease. This shows the importance of considering individual differences to adapt AD diagnosis and personalize early interventions.